# Automatically identifying social isolation from clinical narratives for patients with prostate Cancer

**DOI:** 10.1186/s12911-019-0795-y

**Published:** 2019-03-14

**Authors:** Vivienne J Zhu, Leslie A Lenert, Brian E Bunnell, Jihad S Obeid, Melanie Jefferson, Chanita A Hughes-Halbert

**Affiliations:** 1 Biomedical Informatics Center at Medical University of South Carolina, Chalrleston, South Carolina USA; 2 Holling Cancer Center and Department of Psychiatry and Behavioral Sciences at Medical University of South Carolina, Charleston, South Carolina USA

## Abstract

**Background:**

Social isolation is an important social determinant that impacts health outcomes and mortality among patients. The National Academy of Medicine recently recommended that social isolation be documented in electronic health records (EHR). However, social isolation usually is not recorded or obtained as coded data but rather collected from patient self-report or documented in clinical narratives. This study explores the feasibility and effectiveness of natural language processing (NLP) strategy for identifying patients who are socially isolated from clinical narratives.

**Method:**

We used data from the Medical University of South Carolina (MUSC) Research Data Warehouse. Patients 18 years-of-age or older who were diagnosed with prostate cancer between January 1, 2014 and May 31, 2017 were eligible for this study. NLP pipelines identifying social isolation were developed via extraction of notes on progress, history and physical, consult, emergency department provider, telephone encounter, discharge summary, plan of care, and radiation oncology. Of 4195 eligible prostate cancer patients, we randomly sampled 3138 patients (75%) as a training dataset. The remaining 1057 patients (25%) were used as a test dataset to evaluate NLP algorithm performance. Standard performance measures for the NLP algorithm, including precision, recall, and F-measure, were assessed by expert manual review using the test dataset.

**Results:**

A total of 55,516 clinical notes from 3138 patients were included to develop the lexicon and NLP pipelines for social isolation. Of those, 35 unique patients (1.2%) had social isolation mention(s) in 217 notes. Among 24 terms relevant to social isolation, the most prevalent were “lack of social support,” “lonely,” “social isolation,” “no friends,” and “loneliness”. Among 1057 patients in the test dataset, 17 patients (1.6%) were identified as having social isolation mention(s) in 40 clinical notes. Manual review identified four false positive mentions of social isolation and one false negatives in 154 notes from randomly selected 52 controls. The NLP pipeline demonstrated 90% precision, 97% recall, and 93% F-measure. The major reasons for a false positive included the ambiguities of the experiencer of social isolation, negation, and alternate meaning of words.

**Conclusions:**

Our NLP algorithms demonstrate a highly accurate approach to identify social isolation.

## Background

Social isolation is an important social determinant (SD) that significantly affects patient health outcomes and mortality, with an impact equal to standard clinical factors such as smoking, obesity, and hypertension [1, 2]. Social isolation refers to the extent to which individuals perceive that the quality and quantity of their social relationships are insufficient to meet their social needs [3]. Indicators of social isolation include having limited contact with members of one’s social network, lack of social support, and feeling lonely [4]. Consistent with this, social isolation instruments used in population-based samples ask individuals to self-report the extent to which they lack companionship, feel left out, and feel isolated from others [5]. Social isolation has been associated with an increased risk for cancer recurrence and mortality among breast cancer patients [6]. Recently, Ettridge and colleagues found that prostate cancer patients experience social isolation because of treatment-related side effects (e.g., incontinence) [7]. However, studies have not examined the extent to which social isolation is discussed and documented in electronic health records (EHR) among prostate cancer patients despite recommendations from the National Academy of Medicine to document this issue in electronic clinical notes [8].

Health care systems were advised to collect evidence of SDs such as social isolation prospectively using survey-based data collection tools [9]. While obtaining those data through patient surveys and questionnaires may be the desired method, administering these instruments may interfere with clinical practice and workflow [10]. Unlike other SDs such as race/ethnicity, depression, alcohol use, and nicotine use, information about social isolation is not captured routinely and is usually not encoded in the EHR; however, social isolation might be documented in clinical notes where providers record the information as told by their patients. Because those clinical narratives are available in electronic format, a potential alternative to identify and extract patients’ social isolation information from clinical narratives is natural language processing (NLP). NLP is a technique that uses both grammatical and statistical processes to parse free text and automatically convert those data to a structured format that can be stored in a database and applied in analytics [11]. NLP has been successfully applied for diagnostic, patient safety, clinical decision support, and quality performance reporting [12–14]. Recently, researchers developed NLP strategies to extract SDs from clinical notes, such as substance use, homelessness, and adverse childhood experience [15, 16]; however, NLP approaches have not been developed and evaluated specifically for social isolation. In this project, we developed an NLP approach for extracting information on social isolation from a large dataset of clinical notes. We also formally evaluated the NLP algorithm performance against a gold standard (i.e. domain expert manual review).

## Method

### Study setting

The study setting was the Medical University of South Carolina (MUSC). MUSC is an academic medical science center with inpatient, outpatient, and emergency facilities serving Charleston, South Carolina and surrounding areas. MUSC has had the EpicCare EHR system (Epic Systems Corp., Verona, WI) in place for outpatient care since 2012 and for inpatient care since 2014. A Research Data Warehouse (RDW) copies the Epic data warehouse and serves as the data repository for clinical research. This study tests the feasibility of identifying mentions of social isolation in clinical notes using NLP for a defined population consisting of prostate cancer patients. This study was approved by the MUSC Institutional Review Board.

### NLP software

We used commercial NLP software (Linguamatics I2E version 5.3, Cambridge, United Kingdom) to index, parse, and query each clinical note. Linguamatics I2E (I2E) applies concept-based indexing techniques to identify key words/phrases from text documents and electronically map them to concepts in the Unified Medical Language System (UMLS) Metathesaurus [17]. Then, I2E queries retrieve information for reports, meeting a user-defined set of criteria through a user-friendly interface to define syntactic and semantic representations. In previous work using I2E, we abstracted numerator data for the Group Physician Reporting Option (GPRO) quality measure for fall risk assessment. The NLP algorithm identified 62 (out of 144) patients for whom a fall risk screen was documented only in clinical notes and, thus, was not coded. Manual review confirmed 59 patients as true positives and 77 patients as true negatives. Our NLP approach scored 0.92 for precision, 0.95 for recall, and 0.93 for F-measure [18]. Although the concept of fall risk screening is very different from the concept of social isolation, our experience with I2E ensures the development of accurate NLP algorithms identifying social isolation from clinical notes.

### Data source

This study was conducted by a transdisciplinary center in precision medicine and minority men’s health that is addressing racial differences in prostate cancer risk and outcomes. Prostate cancer is one of the leading causes of cancer among men in the United States, and it disproportionately affects African American men in terms of morbidity and mortality [19]; therefore, this is an important patient population to study with respect to social determinants that increase risk for morbidity and mortality. Previous research has shown that experiencing greater social constraints (e.g., strained relationships with family members and friends) is associated with psychological distress among men who have been diagnosed with prostate cancer [20]. Therefore, this study included racially diverse (African American, white) patients who were 18 years of age or older and diagnosed with prostate cancer (ICD 10 codes: R97.21, D29.1, C61, D40.0) between January 1, 2014 and May 31, 2017. Instead of using all kinds of notes (49 note types) existing for this cohort, we selected the most prevalent note types, and all of them together covered 95% of note sample. NLP pipelines identifying social isolation were developed via extraction of notes on progress, history and physical, consult, emergency department provider, telephone encounter, discharge summary, and plan of care. A de-identified subject-ID was used to link source documents and data across each patient’s records. From 4195 eligible prostate cancer patients, we randomly sampled 3138 patients (75%) as a training dataset with 150,990 notes to develop the lexicon and NLP pipelines to detect social isolation mentions. The remaining 1057 patients (25%) were used as a test dataset, with 55,516 notes used to evaluate NLP algorithm performance.

### Development of the lexicon for social isolation

Generating a lexicon for social isolation is challenging because there are no documented standards, and data collection strategies for social isolation in the EHR are in an early stage of development. We used the Loneliness Scale and domain experts’ knowledge to generate a lexicon that appropriately represents social isolation mentions [5]. Similar concepts are measured in other social isolation instrument; for instance, the social isolation questionnaire in the Patient-Reported Outcomes Measurement Information System (PROMIS) asks respondents to indicate if they feel left out and feel isolated from others [21]. The initial list of terms was provided by behavioral science researchers (CHH and MJ) who have extensive experience in health care quality and disparity research. These terms included “lack companionship”, “feel left out”, “isolated”, “loneliness”, and “lonely”. Using I2E to query these seed terms against the training dataset, the NLP informatics team developed a draft of enhanced terminology set, omit for conciseness covered by these seed terms. The additional terms include “no friend”, “social withdraw”, the combination of “not have reliable/questionable/no/lack”, “family”, and “companionship/support/network”, and the combination of “limited/absence of/work in increasing/lack/loss of/no”, “social”, and “network/support/connection/contact”. During this process, we noticed that the term “live alone” appeared in many patient notes; however, “live alone” doesn’t necessary indicate social isolation and, thus, is not included in the lexicon [22]. For each term, we utilized the I2E morphologic and case variants functions and I2E built-in ontology to generate a set of spelling variants, acronyms, and abbreviations; we then queried these terms against clinical notes to extract any relevant lexical representations iteratively to form an enhanced and refined list. The domain expert and the NLP informatics team reached consensus agreement, creating the final lexicon. To exclude false mentions of social isolation, we utilized I2E built-in pre- and post-negation, using a collection of regular terms that are negative mentions (e.g. “no,” “deny,” “negative”), as well as indications of an historical event or a family member as the experiencer. A final lexicon list was imported to the I2E customized macros, thus allowing for re-use and refinement.

### Development of NLP algorithm to identify social isolation

We developed a set of I2E queries to identify social isolation mentions in clinical notes using the following criteria: a) mentions of social isolation by the lexicon; and b) exclusion of social isolation mentions through negations. These I2E queries were designed to capture semantic information, syntactic patterns, and clinical negations in order to translate a documented social isolation to the following structured data elements: 1) patient medical record number (MRN), 2) social isolation, 3) author type, 4) note ID, 5) date of document, and 6) type of clinical note. We used I2E 5.4 to index, query, flag, and count the number of query hits within each clinical note. We used all clinical notes in the training dataset to develop an NLP algorithm for each variable, and then evaluated the results of the I2E queries independently and in combination against the gold standard of expert chart review. These chart review evaluations were performed independently by a domain expert who was blinded to the I2E query development. Discrepancies between query results and the manual expert review led the informatics team to conduct error analyses and iteratively refine the I2E query algorithms until sensitivity and specificity could not be improved.

### NLP algorithm performance evaluation

Using clinical notes in the test dataset (55,516 notes from 1057 patients), we compared the results generated from the I2E queries to the results from the gold standard. Two reviewers (BB and JO) validated the NLP results by manual chart review. The reviewers were trained regarding the operational definition of social isolation and the methods of NLP assisted chart review. Given a sample size of 1057 patients in the test dataset and 5% prevalence of social isolation in the elderly from another study [23], we estimated a manual review of 69 patients’ notes would achieve a 95% confidence level for the evaluated performance. We anticipated that the rate of NLP identified social isolation positives was similar to that in the training dataset; the reviewer validated chart for all the NLP identified social isolation positives. The reviewers also assessed clinical notes for randomly selected NLP identified negatives for 69 patient patients minus the number of NLP identified positive patients. We calculated three standard performance measures for the NLP algorithm: the precision, recall, and *F*-measure. Precision (exactness) is the proportion of true positives to the total number of algorithm-identified cases; in contrast, recall (completeness) is the proportion of true positives that are retrieved by algorithms [24]. Finally, for all false positives and negatives generated by the NLP algorithms, the reasons for false classification were manually determined and summarized to improve the algorithm.

## Results

### Social isolation lexicon

**Fig. 1:**
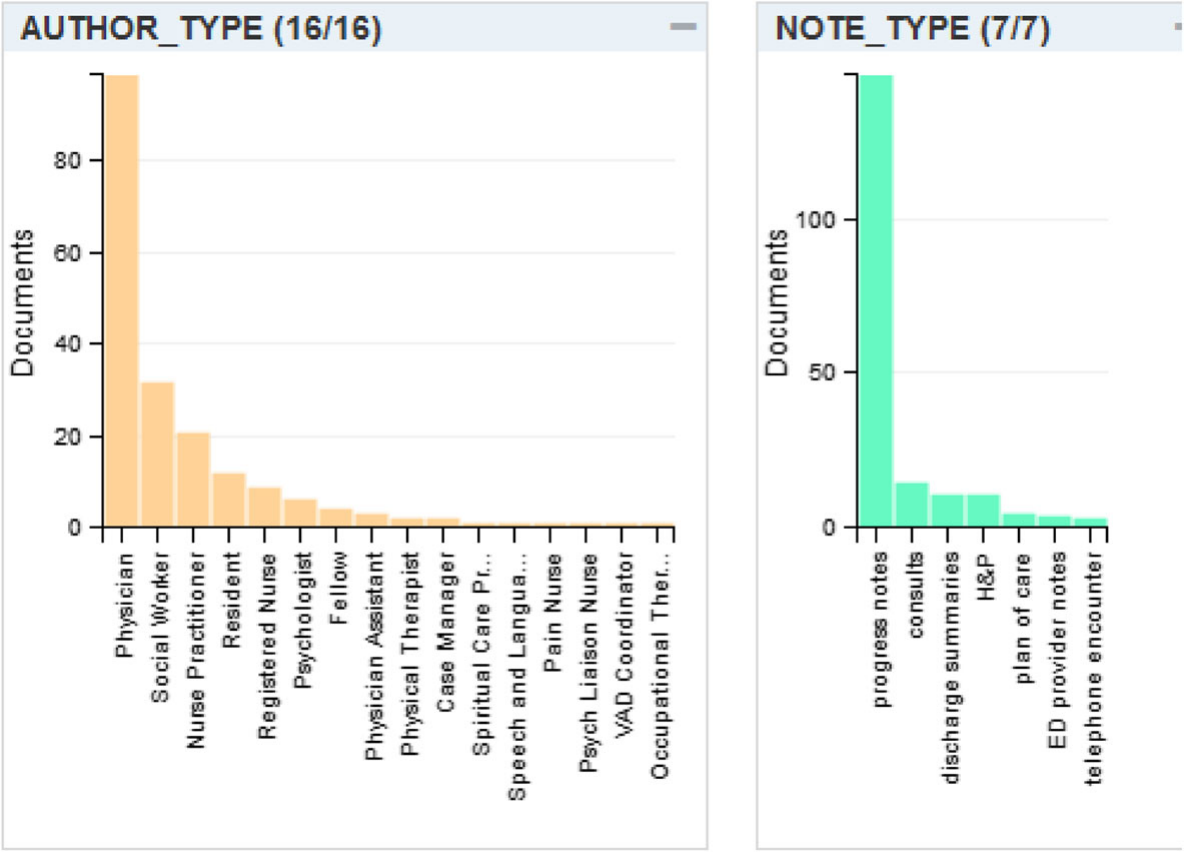
Distribution of located social isolation mentions in different note types and provider types

The I2E multiple query processed the training dataset (150,990 documents from 3138 unique patients) within 8 s. The average number of documents per patient was 48 (max: 994; minimum: 1). The number of each note type is listed in Table 1. We developed a set of I2E queries to search the enhanced key words (e.g. “isolated,” “lonely,” “no friend,” “lack of social support,” etc.) against these documents. After iterative evaluations between the keywords hit and the original documents, we developed a lexicon of social isolation and negations. The final lexicon presented wide variations (Table 2). A total of 24 terms associated with social isolation resulted in 266 hits in 217 documents from 35 unique patients (1.2% of the training sample). The leading keywords (with morphologic variants) were “lack of social support” (59 hits, 24.8%), “lonely” (47 hits, 19.7%), “social isolation” (35, 14.7%), “no friends” (35, 14.7%), and “loneliness” (31, 13.0%). Among eight identified I2E built-in pre-negations, only “her husband” is a true pre-negation (i.e., the experiencer of social isolation is the husband, not the patient); other pre-negations such as “but”, “is still”, and “still has” actually indicated an occurrence of social isolation. The term “all family” is usually a pre-negation in a clinical context (e.g., “all family have drink history”, it doesn’t necessary mean that the patient also has a drink history unless supportive information has been identified); however, it appears in the sentence of “he is estranged from all family and has no friends”, thus representing a true case of “social isolation” and thus a false negation. No post-negations were found. The most common notes with instances of social isolation were progress notes and consult notes, and the most common author types were physician, social worker, nurse practitioner, psychologist, and resident (Fig. 1). The distribution of note types in the test dataset was similar (data not shown). The demographics for the NLP identified social isolation positives and negatives from both the training set and the test dataset are summarized in Table 3. The study cohort (prostate cancer patients) were older (nearly 70 years of age on average) and the majority identified as White and as Medicare/Medicaid beneficiaries. Thus, we observed that the individuals who were identified as having social isolation by NLP were more likely to be White or have Medicare/Medicaid coverage. However, the statistical significance of these observations is uncertain due to the small number of NLP identified social isolation positives and the similar distribution of race and insurance type in the NLP identified negatives.

**Table 1:** Number and prevalence of different note type in training data set

Note type	Number	Percentage
Progress notes	788,18	52.2%
Telephone encounter	41,699	27.6%
Plan of care	19,337	12.8%
Consults	3744	2.5%
H&P	3040	2.0%
Discharge summary	2429	1.6%
ED provider	1923	1.3%
Total	150,990	100.0%

**Table 2:** Lexicon of social isolation and frequency

Terms of social isolation	Frequency		Terms of social isolation	Frequency		I2E Pre-negation	
lack of social support	52	19.5%	Limited social support	3	1.1%	but *	3
lonely	41	15.4%	feel isolated	3	1.1%	all family*	2
no friends	35	13.2%	no family support	3	1.1%	her husband	1
loneliness	29	10.9%	isolation and loneliness	2	0.8%	Discussed*	1
Social withdraw	26	9.8%	Socially withdrawn	2	0.8%	However*	1
socially isolated	22	8.3%	socially isolating	2	0.8%	is still*	1
social isolation	9	3.4%	Social isolation	2	0.8%	still has*	1
feels isolated	8	3.0%	Limited social support	2	0.8%	But*	1
Lonely	6	2.3%	limited social connection	1	0.4%		
lack of social supports	6	2.3%	Limited social network	1	0.4%		
no social support	5	1.9%	lack in social support	1	0.4%		
Loneliness	4	1.5%	loss of social network	1	0.4%		

*false negation

**Table 3:** Demographics for NLP identified positives and negatives of social isolation

	Positives	Negatives
Number of patient (%)	52 (1.2%)	4143 (98.8%)
Age at prostate cancer diagnosis	69.7 ± 9.0	70.2 ± 8.8
Race		
White	37 (1.4%)	2646 (98.6%)
African American	15 (1.1%)	1388 (98.9%)
Other	0 (0%)	109 (100%)
Insurance Type		
Commercial	1 (0.2%)	665 (99.8%)
Medicaid/Medicare	47(4.7%)	2944 (95.3%)
Other	4(0.4%)	951 (99.6%)

### NLP algorithm performance

**Fig. 2:**
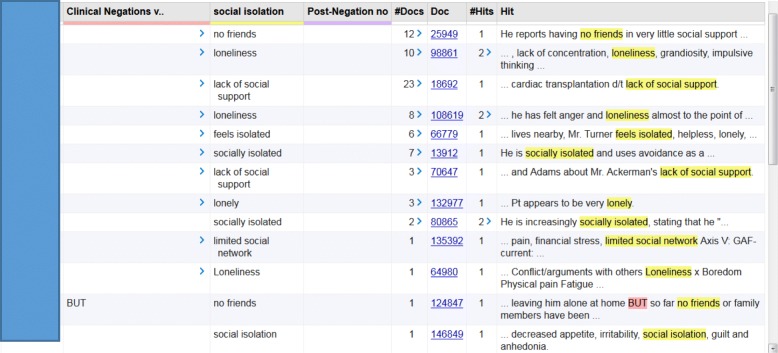
Example of I2E query output

**Fig. 3:**
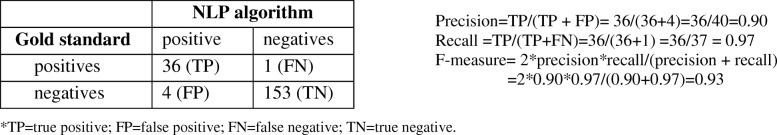
Results of manual review and I2E algorithm

The multiple I2E query combining seven I2E queries produced a structured output table which extracted a patient MRN, keywords for social isolation, the sentences where keywords hit, note type, author type, and note creation date. It also provided a link to the original document, which the NLP developer and reviewers could validate during the development and evaluation phases (Fig. 2). Among 55,516 notes from 1057 patients in the test dataset, I2E query identified 40 notes with a likely mention of social isolation from 17 patients (1.6%). Three patients had a pre-negation identified, such as “risk” and “however”, for the same reason as observed from the training dataset. These patients actually had a social isolation mention in their notes; thus, these mentions with pre-negations were counted as positives of social isolation. Among these 40 NLP identified notes with a social isolation mention, two reviewers’ manual assessment confirmed 36 notes from 13 patients with a social isolation mention and identified four notes as false positives from four patients. Two reviewers also manually evaluated 154 notes for 52 patients randomly selected from controls who had no social isolation mention identified by NLP algorithms and found one false negative. Among 194 notes, the two reviewers agreed on 36 positives and 153 negatives. The inter-rater agreement is 97.4%. Counting on document level, the I2E query for social isolation had a precision of 0.90, recall of 0.97, and an F-measure 0.93 (Fig. 3). The following major reasons accounted for false positives: Our NLP approach could not completely exclude some false social isolation mentions that were applied to a family member, or “No” appeared as the answer after such a mention, or the term of social isolation was part of a group topic, which is not an indication for this study (Table 4).

**Table 4:** Detailed information of false positives of social isolation

Lonely/Isolated X Pt’s wife states that she has very little social support from her community.	
Patients will learn what HALTS stands for (Hungry Angry Lonely Tired Sick)	
Lonely/Isolated?? No	
Pt will require 24 h assistance at discharge however questionable family support.	

### NLP algorithm performance

The multiple I2E query combining seven I2E queries produced a structured output table which extracted a patient MRN, keywords for social isolation, the sentences where keywords hit, note type, author type, and note creation date. It also provided a link to the original document, which the NLP developer and reviewers could validate during the development and evaluation phases (Fig. 2). Among 55,516 notes from 1057 patients in the test dataset, I2E query identified 40 notes with a likely mention of social isolation from 17 patients (1.6%). Three patients had a pre-negation identified, such as “risk” and “however”, for the same reason as observed from the training dataset. These patients actually had a social isolation mention in their notes; thus, these mentions with pre-negations were counted as positives of social isolation. Among these 40 NLP identified notes with a social isolation mention, two reviewers’ manual assessment confirmed 36 notes from 13 patients with a social isolation mention and identified four notes as false positives from four patients. Two reviewers also manually evaluated 154 notes for 52 patients randomly selected from controls who had no social isolation mention identified by NLP algorithms and found one false negative. Among 194 notes, the two reviewers agreed on 36 positives and 153 negatives. The inter-rater agreement is 97.4%. Counting on document level, the I2E query for social isolation had a precision of 0.90, recall of 0.97, and an F-measure 0.93 (Fig. 3). The following major reasons accounted for false positives: Our NLP approach could not completely exclude some false social isolation mentions that were applied to a family member, or “No” appeared as the answer after such a mention, or the term of social isolation was part of a group topic, which is not an indication for this study (Table 4).

## Discussion

This study demonstrated that NLP could extract social isolation information from clinical notes with high precision and recall. To the best of our knowledge, this is the first report of an NLP–based extraction for social isolation. Secondly, our study found that about 1.2% of patients with prostate cancer had social isolation mention(s) in their clinical narratives; this finding is consistent with a prior study, which reported that 1.2% of elderly adults were subjectively isolated (based on an individual’s perceptions about quality of his/her social relationship), and 5% of elderly adults were objectively isolated (through physical separation from and insufficient interaction with other people) [23]. However, other studies have estimated a 15–40% prevalence of social isolation among elderly adults, without reliance on a consensus definition and operationalization of social isolation [25]. Therefore, the true prevalence of social isolation in our study population is uncertain without access to confirmation from other measurements; notably unknown is the extent to which social isolation information is commonly recorded in providers’ clinical notes. Thus, a prospective study assessing concordance between NLP-based identification and survey-based measures of social isolation is needed to evaluate if the NLP approach can accurately reflect patients’ social isolation. Third, a recent NLP study reported that social support was indicated in 0.3–1.4% of clinical notes for a general patient population (in the social history section of providers’ notes) [26]. In the current study, we observed that social isolation information might also be documented in other sections of clinical notes where providers have recorded health issues expressed by patients, especially in the section, “history of present illness.” For example, the following clinical notes: “He feels that he is becoming socially isolated because of his voice issues,” “decreased social interaction .... Some sadness and social isolation” were identified by our algorithm. Our study also found that African Americans have a slightly lower prevalence of social isolation compared to whites; this finding is consistent with previous research which found that social network ties are more prevalent among African Americans [27]. Importantly, previous studies have shown that social isolation is common in elderly patients and is an independent risk factor for mortality. Other significant predictors of mortality in the elderly include older age, male gender, less wealth, and the presence of cancer [28]. Our study found that prostate cancer patients with evidence of social isolation had similar demographic, social, and economic characteristics leading to multiple risk factors for mortality. Thus, in addition to medical treatment, providing sufficient social support to those patients is important to improve their quality of life and survival rate. Additional research is needed to determine the extent to which social isolation identified from EHR using NLP is associated with health care outcomes among this patient population.

Using NLP to identify social isolation in electronic health records poses several challenges. Standard terminology, such as SNOMED-CT and MESH, include a broad array of terms with the full coverage of medical specialties and, thus, can guide data extraction from clinical notes [29]. However, clinical notes are commonly documented as natural language by providers, and examining standard terminology alone may miss important information embedded in clinical notes. For example, in SNOMED-CT social isolation is represented by four concepts: “social isolation,” “social exclusion,” “social withdraw,” and “social outcast”. In our study, we found that only “social isolation” and “social withdraw” are prevalent in clinical notes with a modest frequency. We observed that “social exclusion” and “social outcast” were not found in clinical notes; the plausible explanation is that these two standard SNOMED terms are not commonly used in clinical settings or between/among providers, and thus those terms are not present in the clinical notes. Instead, the most prevalent term we found through our NLP analysis is “lack of social support”, which is not a specific component of the instruments that are used to measure social isolation by self-report; however, lack of social support has been used as an indication of social isolation in previous research [4]. Because the lexicon generated from the current study combines standard concepts and domain expert knowledge, our approach offers a more complete data extraction method. In this study, we utilized I2E’s user-friendly interface tools to accelerate the development of a lexicon for social isolation; the lexicon generated from this study is reusable for open source NLP software (e.g., cTAKES) as customized dictionaries, thus the lexicon establishes a foundation for rapid progress on NLP tasks of social isolation extraction and later dissemination across research communities. Another challenge is that clinical notes do not always follow standard grammar and format. Although our NLP algorithms maximally mimic a common way that providers document social isolation, false positives are inevitable. For example, in “Lonely/Isolated X Pt’s wife states that she has very little social support from her community,” although our NLP algorithms applied negation for another family member as an experiencer, our algorithms could not clearly tell that the experiencer is “Pt’s wife” for “Lonely/Isolated”, or the experiencer is “she” for “little social support”. Also, in “Pt will require 24hour assistance at discharge however questionable family support”, “questionable family support” is a correct semantic representation of social isolation; however, the sentence refers to required care and, thus, does not indicate that the patient lacks family support. Since social isolation is not commonly recorded in clinical notes, and there is no standard diagnostic code to document social isolation in the EHR, we intended to develop a highly sensitive NLP algorithm that avoids false negatives by sacrificing specificity. After iterative evaluation and refinement, our final I2E algorithm achieved 0.90 precision and 0.97 recall, which indicated that our NLP approach could effectively identify social isolation when such information is available in clinical notes.

### Limitations

Some limitations of this study should be noted. First, this study only included prostate cancer patients at a single academic institution. Our lexicon and NLP algorithms were developed to reflect the definition of social isolation for prostate cancer patients and may miss other terms suitable for other populations; therefore, the lexicon and algorithm s may not be generalizable to other populations with different diseases or to other institutions without customization and evaluation. Second, the lexicon in this study may not include all potential synonymous variants of social isolation, and there may be conceptual overlap with some of the terms included in our lexicon. Because there is no standard terminology for documenting social isolation in the EHR, we also included terms (e.g., social support) that have been used to indicate social isolation in previous research in order to in order to develop an inclusive lexicon [4]. Previous research has shown that “lack of social support” and “social isolation” are associated with each other and have similar effects on patients’ outcomes [30, 31]. However, social support may be documented in the EHR as the patient’s perception of the level of instrumental, emotional, tangible, or emotional support provided by others. Third, we utilized I2E built-in negations; however, we observed that some negations identified from clinical notes were not true negations within the context of social isolation identification, such as “Discussed,” “However,” “but”. Although these built-in negations may accurately exclude false positives for other domains, a customized negation should be generated for better capture of social isolation mention(s). Finally, our NLP approach could extract social isolation from clinical notes with high accuracy; however, the clinical notes typically capture information about providers’ observations about patients and some patients’ chief complaints. Social isolation information may be under-documented in clinical notes. Therefore, we remain cautious about claiming an NLP determinant for prevalence of social isolation (1.2%) in patients with prostate cancer, due to the lack of confirmation from other measures. To understand whether a patient experiences social isolation, a more comprehensive assessment is warranted.

## Conclusions

Our NLP approach demonstrate a highly accurate approach to identify social isolation when such information is available in clinical notes. However, the lexicon was specifically developed for prostate cancer patients. Thus, customization and evaluation are needed for studying social isolation NLP extraction for other populations.

## Abbreviations

EHR: electronic health records
GPRO: Group Physician Reporting Option
I2E: Linguamatics I2E
IRB: Institutional review Board for Human Research
MRN: medical record number
MUSC: Medical University of South Carolina
NLP: natural language processing
PROMIS: Patient-Reported Outcomes Measurement Information System
RDW: Research Data Warehouse
SD: social determinant
UMLS: Unified Medical Language System
