# Correction to: Automatically identifying social isolation from clinical narratives for patients with prostate Cancer

**DOI:** 10.1186/s12911-019-0815-y

**Published:** 2019-04-25

**Authors:** Vivienne J. Zhu, Leslie A. Lenert, Brian E. Bunnell, Jihad S. Obeid, Melanie Jefferson, Chanita Hughes Halbert

**Affiliations:** 1 Biomedical Informatics Center, Medical University of South Carolina, Chalrleston, SC USA; 2 Holling Cancer Center and Department of Psychiatry and Behavioral Sciences, Medical University of South Carolina, Charleston, SC USA

**Correction to: BMC Med Inform Decis Mak**

**https://doi.org/10.1186/s12911-019-0795-y**

Following publication of the original article [1], the authors reported an error in one of the authors’ names. In this Correction the incorrect and correct author name are shown. The original publication of this article has been corrected.

Originally the author name was published as:

- Chanita A Hughes-Halbert

The correct author name is:

- Chanita Hughes Halbert
